# Transcriptional Regulation of Cystathionine-γ-Lyase in Endothelial Cells by NADPH Oxidase 4-Dependent Signaling*

**DOI:** 10.1074/jbc.M115.685578

**Published:** 2015-11-30

**Authors:** Rajesh K. Mistry, Thomas V. A. Murray, Oleksandra Prysyazhna, Daniel Martin, Joseph R. Burgoyne, Celio Santos, Philip Eaton, Ajay M. Shah, Alison C. Brewer

**Affiliations:** From the ‡ Cardiovascular Division, King's College London British Heart Foundation Centre, 125 Coldharbour Lane, London SE5 9NU and; § Cardiovascular Division, King's College London British Heart Foundation Centre, The Rayne Institute, St. Thomas' Hospital, London SE1 7EH, United Kingdom

**Keywords:** endothelial cell, hydrogen sulfide, reactive oxygen species (ROS), redox signaling, transcription regulation, transgenic mice, ATF4, CSE, NADPH oxidase 4, vascular tone

## Abstract

The gasotransmitter, hydrogen sulfide (H_2_S) is recognized as an important mediator of endothelial cell homeostasis and function that impacts upon vascular tone and blood pressure. Cystathionine-γ-lyase (CSE) is the predominant endothelial generator of H_2_S, and recent evidence suggests that its transcriptional expression is regulated by the reactive oxygen species, H_2_O_2_. However, the cellular source of H_2_O_2_ and the redox-dependent molecular signaling pathway that modulates this is not known. We aimed to investigate the role of Nox4, an endothelial generator of H_2_O_2_, in the regulation of CSE in endothelial cells. Both gain- and loss-of-function experiments in human endothelial cells *in vitro* demonstrated Nox4 to be a positive regulator of CSE transcription and protein expression. We demonstrate that this is dependent upon a heme-regulated inhibitor kinase/eIF2α/activating transcription factor 4 (ATF4) signaling module. ATF4 was further demonstrated to bind directly to *cis*-regulatory sequences within the first intron of CSE to activate transcription. Furthermore, CSE expression was also increased in cardiac microvascular endothelial cells, isolated from endothelial-specific Nox4 transgenic mice, compared with wild-type littermate controls. Using wire myography we demonstrate that endothelial-specific Nox4 transgenic mice exhibit a hypo-contractile phenotype in response to phenylephrine that was abolished when vessels were incubated with a CSE inhibitor, propargylglycine. We, therefore, conclude that Nox4 is a positive transcriptional regulator of CSE in endothelial cells and propose that it may in turn contribute to the regulation of vascular tone via the modulation of H_2_S production.

## Introduction

The importance of the endothelium within the cardiovascular system is highlighted by the many diseases that arise from its dysfunction, including atherosclerosis, hypertension, and metastatic disease (1). The endothelium acts to regulate vascular tone through the production and release of multiple vasoactive agents, including the gasotransmitters nitric oxide (NO) (2), carbon monoxide (CO) (3), and hydrogen sulfide (H_2_S) (4). These gasotransmitters directly modify target cellular proteins and act by diverse mechanisms to exert their function (5). For instance, it is well established that NO acts directly upon soluble guanylate cyclase in adjacent vascular smooth muscle cells to increase cyclic GMP levels (5). H_2_S is the most recently identified and least understood of these physiologically relevant gasotransmitters. Unlike NO, H_2_S appears to function as an endothelium-derived hyperpolarizing factor, which acts upon potassium channels both within the endothelium itself, and the adjacent vascular smooth muscle cells (4). In the case of the ATP-sensitive potassium channel (within vascular smooth muscle cells) a mechanism involving activation via *S*-sulfhydration of a specific cysteine residue has been demonstrated (4). H_2_S is synthesized endogenously within vascular cells, including endothelial cells, through the action of cystathionine γ-lyase (CSE),^2^ from a number of substrates including cysteine, cystine, and homocysteine (6). The physiological importance of CSE-generated H_2_S has been demonstrated, as CSE-null mice display pronounced hypertension and impaired vascular relaxation to acetylcholine (7). Being gaseous and highly reactive, H_2_S is not readily stored in vesicles and hence must be synthesized, on demand, close to its site of action. Consequently both the expression and activity of CSE must be tightly controlled.

An emerging body of evidence suggests that the regulation of CSE expression is subject to redox-dependent mechanisms and is promoted in response to a pro-oxidative cellular environment. Thus the administration of exogenous H_2_O_2_ was shown to up-regulate CSE gene expression in fibroblasts (8). Moreover, in mesangial cells, CSE expression was increased by the administration of platelet-derived growth factor-BB, and this increase was significantly reduced by the co-administration of diverse anti-oxidants (9). Although difficult to quantify (10), H_2_S production has recently been demonstrated to be dependent upon H_2_O_2_ using a novel cell-trappable fluorescent probe, allowing real-time visualization of H_2_S. Thus treatment of human umbilical vein endothelial cells (HUVECs) with vascular endothelial growth factor resulted in a CSE-dependent increase in H_2_S production that could be abolished by the addition of the H_2_O_2_ scavenger, catalase, or diphenyleneiodonium chloride, a broad-spectrum inhibitor of the ROS-generating NADPH oxidases (and other flavoproteins) (11).

Although these experiments suggest a role for ROS and, in particular, H_2_O_2_ in the regulation of CSE, the biologically relevant cellular source(s) of ROS and the redox-dependent molecular pathway(s) that underlies this regulation remain unknown. The inhibition of this process by diphenyleneiodonium chloride is, however, suggestive of the involvement of NADPH oxidase(s). NADPH oxidases comprise a family of enzymes whose primary physiological function is the production of ROS, which mediates redox-dependent molecular signaling pathways (12). Their physiological and pathophysiological roles in the cardiovascular system have been extensively studied (13, 14), and within the endothelium NADPH oxidase 4 (Nox4) is the most abundantly expressed isoform. Nox4 has been further shown to participate in the regulation of vascular tone *in vivo* in both gain- and loss-of-function mouse models. Thus we have demonstrated previously that endothelial-specific Nox4-overexpressing transgenic (eNox4 Tg) mice display significantly greater acetylcholine-induced vasodilatation and lower blood pressure compared with wild-type (WT) littermate controls (15), whereas in a separate study a decrease in phenylephrine-induced contraction was demonstrated in vessels isolated from a similar mouse model (16). In addition, it has been demonstrated that (global) ablation of Nox4 results in impaired aortic vascular relaxation to acetylcholine after treatment with angiotensin II (17). These beneficial, protective effects of Nox4 within the vasculature are in marked contrast to the endothelial dysfunction reported to be promoted by both Nox1 and Nox2 (18, 19). This difference is perhaps explained by the fact that Nox4 predominantly generates H_2_O_2_, whereas Nox1 and Nox2 are known to generate the superoxide (O_2_^˙̄^), which reacts with (and hence reduces the bioavailability of) NO within the vasculature (20). Alternatively, a distinct pattern of subcellular localization of Nox4 may couple the ROS it generates to different downstream signaling cascades (21). The aim of this study was to investigate the relationship between Nox4-dependent signaling and CSE expression in endothelial cells.

## Experimental Procedures

### 

#### 

##### Mice

All procedures were performed in accordance with the Guidance on the Operation of the Animals (Scientific Procedures) Act, 1986 (UK Home Office). Generation of eNox4 Tg mice has been described before (15). Tg lines were maintained on a C57Bl6/j background by Wt/Het crosses. 8–10-week male mice were used for wire myography studies.

##### Cell Culture

HUVECs (Lonza) seeded onto precoated dishes (0.4% gelatin in PBS) were maintained in EBM-2 HUVEC media (Lonza). Nox4 overexpression was performed by adenoviral transduction (AdNox4 or Adβ-gal control as described previously (22) using a multiplicity of infection of 20 unless otherwise stated. Transient transfections of (control) pCDNA3.1 (Invitrogen) or an equivalent plasmid in which the full-length mouse ATF4 cDNA had been inserted (pATF4; Addgene) were carried out using the turbofect reagent (Thermo Scientific) following the manufacturers' specifications using 4 μg of plasmid DNA/6-cm dish and incubated for 24 h. For silencing experiments, HUVECs were cultured in 6-cm dishes and serum-starved for 1 h in Opti-MEM before being transfected with siRNAs targeted to Nox4 (5 nm; s27015), p22^Phox^ (20 nm; s194371), ATF4 (20 nm; s1702), heme-regulated inhibitor kinase (HRI; 20 nm; s25823), protein kinase R-like ER kinase (PERK; 20 nm; s18101), or scrambled control siRNA at equivalent concentrations (all Ambion Silencer-select) and incubated for 24 or 48 h as indicated. Transfections were performed using the Lipofectamine 2000® transfection reagent kit (Invitrogen) according to the manufacturers' specifications. For tunicamycin treatments, HUVECs were cultured in 6-cm dishes and treated with 2 μg/ml tunicamycin (Sigma) for 2 h. For H_2_O_2_ treatment, HUVECs were cultured in 6-cm dishes and treated with 10 mm H_2_O_2_ (Sigma) for 1 h. For experiments involving the inhibition of heme biosynthesis and degradation, HUVEC were cultured in 6-cm dishes and treated with either 1 mm oxoheptane (4,6-dioxoheptanoic acid) (Sigma), 10 μm tin protoporphyrin (Sigma), or DMSO vehicle control. For hypoxia experiments, HUVECs were cultured in 6-cm dishes and incubated at either 21% O_2_ in a standard tissue culture incubator or at 1% O_2_ in a hypoxic chamber (Biospherix C Chamber) for 1 h. For assessment of proliferation, HUVEC were treated with Nox4-targeted or control siRNA for 24 h (as above) before being counted in an automated cell counter (TC20, Bio-Rad) and diluted to 125,000 cells/ml. 100 μl of diluted cells were then placed into individual wells of 16-well E-plates (ACEA). Immediately after plating the cell index was measured over 24 h using an xCELLigence real time cell analyzer (RTCA DP, ACEA). In additional experiments, to determine cell viability, cells were supplemented after 5 h with 1 mm dimethyloxalylglycine.

Human embryonic kidney 293 (HEK293) were grown in DMEM (D6546; Sigma) supplemented with 10% fetal bovine serum and 1% penicillin/streptomycin/glutamine. For luciferase assays, HEK cells were transfected in 24-well plates with a constant amount of 900 ng of total DNA composed of 300 ng of test plasmid, 300 ng of reference plasmid (pRL-TK, Promega), and 300 ng of pCDNA3.1 or pATF4 using the TurboFect transfection reagent (2 μl/well; Thermo Scientific).

##### Luciferase Assays

Cells were lysed after 24 h, and activity was assessed using Dual-Glo® Luciferase Assay System (Promega) according to the manufacturers' instructions. In all cases the relative luciferase activity of the test plasmid is shown, comparing the activity of the test plasmid to that of the co-transfected reference plasmid, pRL-TK, and represents the average of triplicate biological samples.

##### Chromatin Immunoprecipitations (ChIP Assays)

ChIP assays were performed using the SimpleChIP enzymatic chromatin IP kit (Magnetic Beads; Cell Signaling). Briefly, T175 tissue culture flasks containing ∼4 × 10^7^ HEK cells were transfected with 25 μg of DNA with 50 μl of Lipofectamine. The DNA was composed of 12.5 μg of −764bpluc-IE and 12.5 μg of either pCDNA3.1 (Invitrogen) or pATF4. Cells were incubated overnight in 25 ml of culture medium and subsequently cross-linked by the addition of 675 μl of 37% formaldehyde (10 min at room temperature) before the addition of 2.5 ml of 10× glycine (provided in the kit) and further incubation for 5 min. Subsequent steps were performed exactly as per the manufacturers' instructions. 10 μg of chromatin was used for each immunoprecipitation. Immunoprecipitations were performed using positive control antibody to histone H3 (10 μl), negative control normal rabbit IgG (1 μl; both provided in the kit), or antibody to ATF4 (5 μl; D4B8, Cell Signaling). PCR conditions used to amplify site A and site B were 95 °C for 2 min then 23 cycles at 95 °C for 15 s, 55 °C for 15 s, 72 °C for 30s followed by 72 °C for 7 min. PCR conditions used for negative control RPL30 primers (provided in the kit) were 95 °C for 2 min, then 28 cycles at 95 °C for 15 s, 62 °C for 15 s, 72 °C for 30 s followed by 72 °C for 7 min. All PCR reactions were performed in 20-μl reaction volumes using REDTaq® ReadyMix^™^ PCR Reaction Mix (Sigma) and final primer concentrations of 200 nm (see Table 1 for primer sequences).

**TABLE 1:** **A list of the primers used to measure gene expression in HUVECs and CMECs as well as primers used for cloning** Prox Enh, proximal enhancer.

Primer name	Forward (5′-3′)	Reverse (5′-3′)
**qPCR**		
CSE (human)	ACACTTTTATGTCACCATATTTCCAG	TGTTGCAGAATACATAGAAATATCAGC
CSE (mouse)	GAGGATGAACAGGACCTTCTT	CAGCTTTGACTCGAACTTTTAAGG
ATF4 (human)	TCTCCAGCGACAAGGCTAA	CAATCTGTCCCGGAGAAGG
Nox1 (human)	AAGGATCCTCCGGTTTTACC	TTTGGATGGGTGCATAACAA
Nox2 (human)	CATTCAACCTCTGCCACCAT	CCCCAGCCAAACCAGAAT
Nox4 (human)	TCCTCGGTGGAAACTTTTGT	CCACAACAGAAAACACCAACT
Nox4 (human/mouse)*^a^*	GCCAACGAAGGGGTTAAACA	TGGCCCTTGGTTATACAGCA
PHD4 (human)	ACTTCATCCGAACCCTCAGC	GCGCTGTAACCCCTTCATCT
PHD4 (Rat)	ACCTGGCACAGATGAAAGG	CATCCCCCATGCAACGAGTA
XBP1 (human)	CCTTGTAGTTGAGAACCAGGAG	GGTCCAAGTTGTCCAGAATGC
HRI (human)	CCACTTCGTTCAAGACAGGTG	GCTAAACTCGTCACTACAAGTGAAA
β-A-ctin (human)	GCGAGAAGATGACCGAGATCA	TCACCGGAGTCCATCACGAT
β-Actin(mouse)	CTGTCGAGTCGCGTCCACCC	ATGCCGGAGCCGTTGTCGAC
β−Actin (rat)	CGTGAAAAGATGACCCAGATCA	TGGTACGACCAGAGGGATACAG

**Cloning**		
6414F	AGAGAGGGTACCTGAAGTATGCTGCCCCTC	
764F	AGAGAGGGTACCCTTTTAGGAAGCTGCCAG	
p191R		AGAGAGCTCGAGAGAAGAAGAGAGGAAAAGAACAC
CSE Prox Enh	TTAATGGATCCGTGAGCTGGGTCTGTCTG	TTAATGGATCCCAGAGGTGAATCACCTGAG

*^a^* Used to detect both mouse and human Nox4 in Nox4 overexpression experiments.

##### Gneration of Promoter-luciferase Reporter Clones

Genomic CSE promoter fragment were generated by PCR with Herculase II Fusion DNA Polymerase (Stratagene) using a Pac clone (RP11–42O15, obtained from BACPAC Resources Center, CHORI) as the template. The forward primers (6414F and 764F) incorporated a KpnI restriction within their 5′ ends, whereas the common reverse primer (+191R) incorporated an XhoI restriction site. KpnI/XhoI-digested fragments were subcloned into the KpnI-XhoI cloning sites of pGL4.22 (Promega) to generate −6414bp-luc and −764bp-luc. The 2.4-kb intronic enhancer fragment was generated using the primers CSE Prox Enh F/R, which had BamHI sites incorporated into their 5′ ends. After digestion with BamHI, this fragment was inserted into the unique BamHI site downstream of the SV40 late poly(A) signal in −764-bp luc to generate −764bp-luc-IE. See Table 1 for sequences of cloning primers.

##### Endothelial Cell Isolation

50 μl of streptavidin-coated dynabeads (Invitrogen) were washed 5 times in HBSS and incubated at room temperature with biotinylated antiCD31 antibody (BAF3628, R&D Systems; 50 μg/ml) for 1 h on a tube rotator then washed 3 times in HBSS. Two to three hearts from age-matched WT and eNox4 Tg mice were pooled according to genotype and finely chopped into 2-mm pieces in HBSS. HBSS was removed, and heart pieces were washed twice more with HBSS to remove blood before digestion in 10 ml of HBSS containing 100 mg/ml collagenase A (10103578001, Roche Applied Science) at 37 °C in a shaker for 20 min. Collagenase A solution was removed and supplemented with 1 ml of calf serum. A fresh solution of HBSS containing collagenase A was added to the remaining heart pieces. This process was repeated twice more. Digests were pooled, passed through a 2-μm cell strainer, and centrifuged at 1200 × *g* for 5 min. Cells were resuspended in 450 μl of HBSS, added to the previously prepared antiCD31-conjugated dynabeads, and placed on a tube rotator for 3 h at room temperature. After this any unbound cells were removed by washing three times with HBSS. Cells bound to the beads were then lysed in 700 μl of qiazol reagent (for RNA analyses; Qiagen) or lysed directly into 50 μl of protein lysis buffer.

##### RNA Analyses

RNA was prepared from tissue-cultured cells using the SV Total RNA Isolation System (Promega) and from primary mouse endothelial cells using the RNeasy Mini kit (Qiagen). cDNA generation and QPCR were performed as described previously (23). Primers used in QPCR reactions are shown in Table 1. XBP-1 spicing was assessed by PCR using primers as described in Table 1 on cDNA generated from HUVEC overexpressing Nox4 for 24 h. PCR reactions were set up using RedTaq ready mix™ and run as follows: 35 cycles of denaturation (30 s at 94 °C), annealing (30 s at 56 °C), and extension (30 s at 72 °C). The resultant PCR products were separated on 1% agarose gels.

##### Western Blot

Protein samples were prepared from cultured cells or homogenized tissue, separated on SDS-PAGE gels, transferred to membranes, and probed with appropriate antibodies (see Table 2) as described previously (22). Generation of the Nox4 antibody has been described previously (24).

**TABLE 2:** **A list of primary and secondary antibodies and their respective dilutions**

Primary antibody	Dilution	Species	Company
CSE	1 in 3000	Rabbit (polyclonal)	Aviva
Nox4	1 in 2000	Rabbit	In house
ATF4 (CREB-2)	1 in 2000	Rabbit (polyclonal)	Santa Cruz
p22^Phox^	1 in 2000	Rabbit (polyclonal)	Abcam
Total eIF2α	1 in 2000	mouse (monoclonal)	Santa Cruz
Pi-eIF2α (Ser51)	1 in 2000	Rabbit (polyclonal)	Millipore
ATF6	1 in 2000	Rabbit	Sigma
β-Actin	1 in 5000	Rabbit	Sigma
IgG-HRP linked	1 in 5000	Goat anti-rabbit	Cell Signaling Technology
IgG-HRP linked	1 in 5000	Horse anti-mouse	Cell signaling Technology

##### Isolation of Mouse Aortae for Wire Tension Myography

8–10-Week-old male mice (eNox4 Tg or WT littermates) were injected intraperitoneally with 45 mg/kg pentobarbital mixed with heparin (1000 IU/kg). The thoracic aorta was removed and immersed in cold (4 °C) Krebs-Ringer solution (2.5 mm CaCl_2_·2H_2_0, 118 mm NaCl, 11 mm glucose d(+), 25 mm NaHCO_3_, 4.7 mm KCl, 1.18 mm KH_2_PO_4_, 1.16 mm MgSO_4_). Aortae were cut into 3-mm-long rings and were mounted in a Danish Myo Technology wire tension myograph. Rings were bathed in Krebs-Ringer solution at 37 °C and supplied with a continuous gas mixture of 95% O_2_ and 5% CO_2_. Before treatment vessels were stretched to the optimal pre-tension condition (∼2 millinewtons) using the Danish Myo Technology normalization modules. Rings were incubated for 30 min in Krebs before the addition of 20 mm propargylglycine (PPG) for 30 min. Vehicle controls composed of Krebs-Ringer solution were run alongside PPG-treated vessels. This was then followed by a cumulative dose-dependent contraction to PE up to 30 μm. Results were analyzed using LabChart software.

##### Statistics

Data are expressed as the means ± S.E. Statistical analyses were performed using unpaired Student's *t* test and by one- or two-way analysis of variance as indicated. *p* < 0.05 using Tukey's post hoc test or Student's *t* test was considered significant.

## Results

### 

#### 

##### Nox4 Is a Physiologically Relevant Positive Regulator of CSE Transcription in HUVECs

**FIGURE 1.**
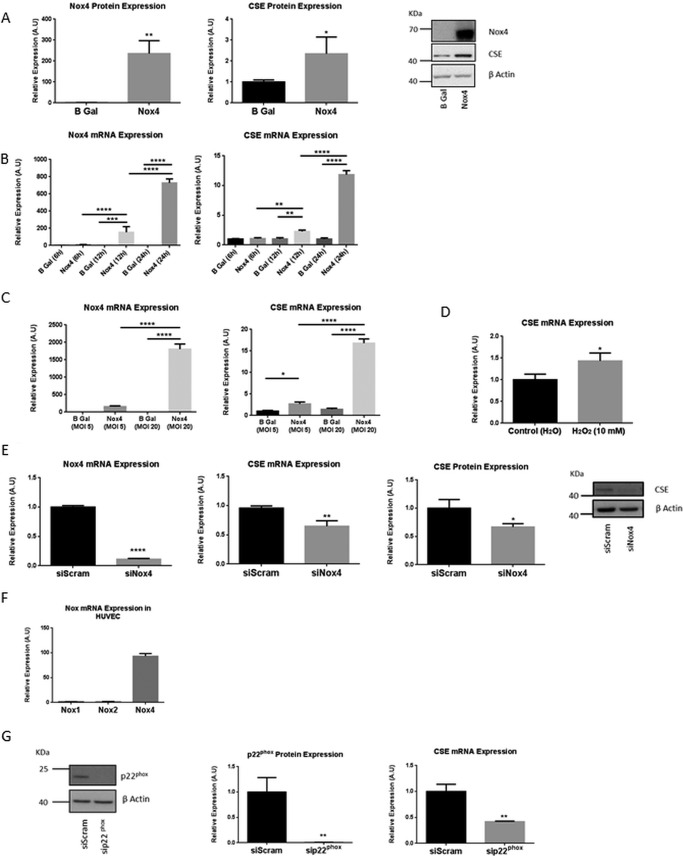
**Nox4 regulated CSE expression in endothelial cells.** *A*, representative Western blot and quantitative histograms indicating Nox4 and CSE protein expression in HUVECs after 48-h Nox4 or β-gal (*B Gal*) overexpression. *B* and *C*, QPCR analyses of Nox4 and CSE mRNA expression after Nox4 or β-gal overexpression in HUVEC for times and multiplicity of infection doses as indicated. *D*, QPCR analyses of CSE mRNA expression in HUVEC after a 10 mm H_2_O_2_ or control H_2_O treatment for 1 h. *E*, QPCR analyses of Nox4 and CSE mRNA expression in HUVECs after 24 h of treatment with Nox4-targeted siRNA (*siNox4*) or control siRNA (*siScram*) and quantitative histogram and representative Western blot depicting CSE protein expression in HUVEC after 48 h of treatment with siNox4 or control siScram. *F*, QPCR analyses of relative expression of Nox1, Nox2, and Nox4 mRNA in HUVECs. *G*, representative Western blot and quantitative histogram indicating p22^phox^ protein expression and corresponding QPCR analyses of CSE mRNA expression in HUVEC after 24 h of treatment with siRNA targeted to p22^phox^ (*sip22^phox^*^)^ or control siRNA (*siScram*). All data normalized to β-actin protein or mRNA expression. *n* = 3 in all cases. *, *p* < 0.05; **, *p* < 0.01; ***, *p* < 0.001; ****, *p* < 0.0001 *A.U.*, absorbance units.

We (and many other groups) have shown previously that overexpression of Nox4 in HUVECs results in an increase in H_2_O_2_ generation (15). To investigate whether Nox4-dependent redox signaling might participate in the regulation of CSE expression in endothelial cells, Nox4 was mis-expressed in HUVECs. Adenovirus-mediated ectopic expression of Nox4 (AdNox4) resulted in a significant and robust up-regulation of both CSE mRNA and protein (Fig. 1, *A* and *B*). This increased accumulation of CSE mRNA upon Nox4 overexpression was found to be both time- and dose-dependent (Fig. 1, *B* and *C*). The efficacy of ROS in the activation of CSE in HUVEC was also demonstrated, as exogenous administration of H_2_O_2_ resulted in a significant increase in CSE expression after 1 h (Fig. 1*D*).

Conversely, siRNA-mediated knockdown of Nox4 in HUVECs resulted in a significant decrease in both CSE mRNA and protein (Fig. 1*E*). The membrane-associated protein, p22^phox^ is an obligate partner of vascular Noxs in the generation of ROS (20), and Nox4 represents by far the most abundant Nox isoform expressed in HUVEC (Fig. 1*F* and Ref. 25). siRNA-mediated down-regulation of p22^phox^ expression similarly acted to reduce CSE expression significantly (Fig. 1*G*). Taken together, these data, therefore, suggest a physiological role for Nox4-dependent signaling in the regulation of CSE transcription in endothelial cells.

##### Nox4 Regulates CSE Expression and Vascular Tone in Isolated Aortic Vessels

**FIGURE 2.**
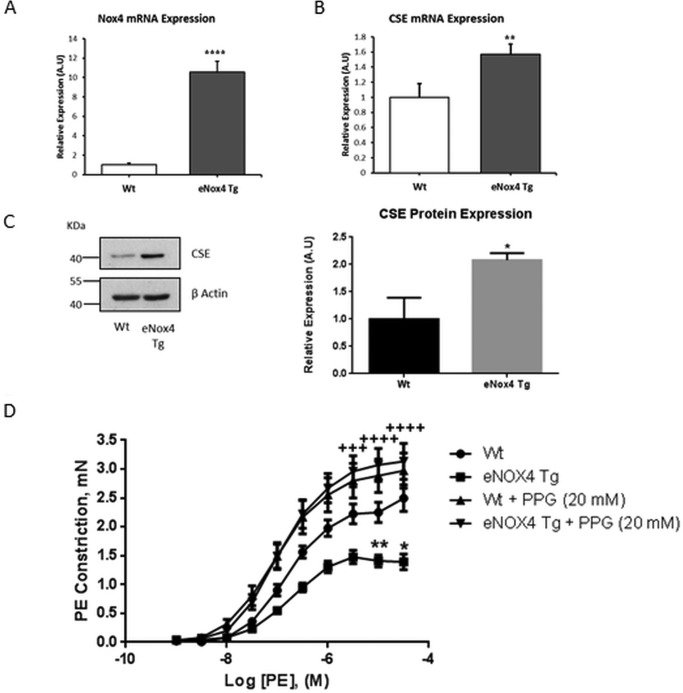
**Nox4 regulated CSE expression in CMECs leading to reduced PE-induced aortic constriction.** *A* and *B*, QPCR analyses of Nox4 and CSE mRNA expression in CMECs isolated from WT and eNox4 Tg mice. *C*, representative Western blot and corresponding densitometric analysis of CSE protein expression in CMECs from WT and eNox4 Tg mice. For *A*, *B*, and *C*, data are representative of 3 separate isolations, where *n* = 2/3/4 in each group in each isolation. *A.U.*, absorbance units. *D*, PE-induced constriction of aortae isolated from WT or eNox4 Tg mice, preincubated with or without 20 mm PPG for 30 min. All data normalized to β-actin mRNA and protein expression. *, *p* < 0.05; **, *p* < 0.01; ****, *p* < 0.0001. Myography data: WT *n* = 8 (12 rings), eNox4 Tg *n* = 6 (9 rings), WT + PPG *n* = 8 (13 rings), eNox4 Tg + PPG *n* = 9 (16 rings). *, *p* < 0.05; **, *p* < 0.01 (* compares WT with eNox4 Tg); +++, *p* < 0.001; ++++, *p* < 0.0001 (+ compares eNox4 Tg with eNox4 Tg + PPG).

We reported previously on the eNox4 Tg mouse line (15). Here we sought to determine whether the Nox4-induced increase in CSE expression demonstrated *in vitro* is maintained in the endothelium of eNox4 Tg mice. Cardiac microvascular endothelial cells (CMECs) were isolated from eNox4 Tg mice and WT littermate controls and analyzed for CSE mRNA and protein expression. The level of transcriptional overexpression of Nox4 in the endothelial cells of these Tg mice was ∼10-fold compared with WT levels (Fig. 2*A*). Consistent with our *in vitro* findings, Nox4 overexpression in endothelial cells *in vivo* similarly resulted in significantly increased CSE mRNA and protein expression (Fig. 2, *B* and *C*).

We next sought to determine the phenotypic consequence of the Nox4-induced increase in CSE expression. CSE has been demonstrated to be the biologically significant generator of H_2_S within vascular endothelial cells (4). Because H_2_S is a gasotransmitter, which acts to regulate vascular tone, and has been reported to have the properties of an endothelium-derived hyperpolarizing factor, we investigated the *ex vivo* vascular function of eNox4 Tg and WT littermates using a wire myograph. Aortic rings isolated from eNox4 Tgs were found to be hypo-contractile compared with WT littermates in response to increasing PE concentrations (Fig. 2*D*). Moreover, in the presence of PPG, an inhibitor of CSE activity, this difference was completely ablated (Fig. 2*D*), consistent with increased CSE expression (and hence H_2_S production) in these mice.

##### Nox4 Up-regulates CSE Expression in HUVECs via Activation of ATF4

**FIGURE 3.**
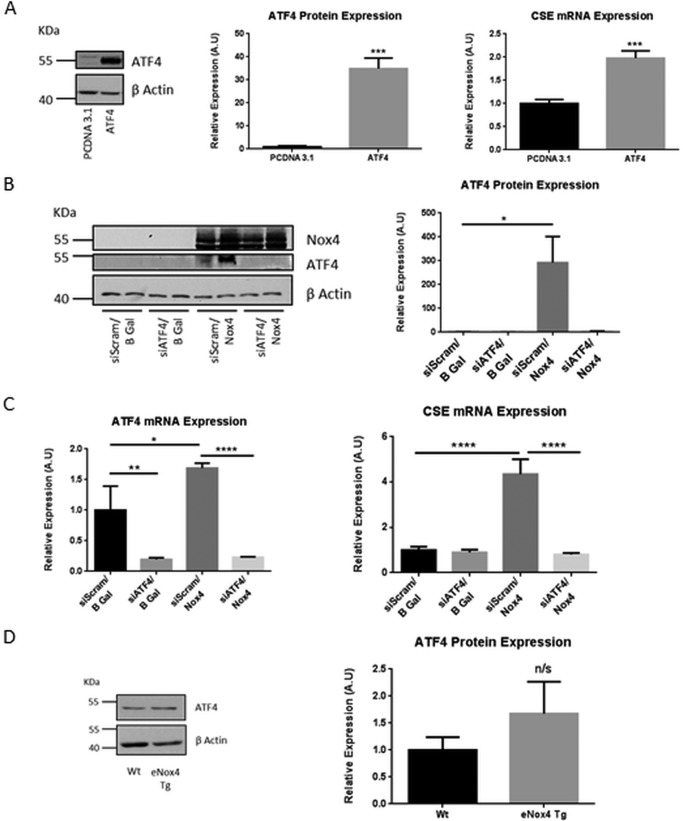
**Nox4 regulated CSE expression via ATF4.** *A*, representative Western blot and quantitative densitometric analyses of ATF4 protein expression and the corresponding QPCR analyses of CSE mRNA expression in HUVECs after 24-h ATF4 or control pCDNA3.1 overexpression. *A.U.*, absorbance units. *B*, representative Western blot and quantitative densitometric analyses of ATF4 protein expression in HUVEC after 48 h of treatment with ATF4-targeted siRNA (siATF4) or control siRNA (siScram) together with 24 h Nox4 or β-gal (*B Gal*) overexpression as indicated. *C*, QPCR analyses of ATF4 and CSE mRNA expression in HUVECs after treatments as in *B. D*, representative Western blot and corresponding densitometric analyses of ATF4 protein expression in CMECs isolated from WT and eNox4 Tg mice. All data are normalized to β-actin mRNA and protein expression. *n* = 3; *, *p* < 0.05; **, *p* < 0.01; ***, *p* < 0.001; ****, *p* < 0.0001.

Nox4 regulates the mRNA expression of CSE indicating the involvement of a transcriptional mechanism in this process. A previous study demonstrated the requirement for the stress-responsive transcription factor, ATF4, in the induction of CSE expression in mouse embryonic fibroblasts (26). Furthermore, the regulation of the activity of ATF4 has been associated previously with Nox4-dependent signaling in cardiomyocytes (27). Overexpression of ATF4 in HUVECs resulted in a highly significant up-regulation in CSE transcription (Fig. 3*A*), suggesting that in endothelial cells, as in mouse embryonic fibroblasts, CSE is a transcriptional target of ATF4. To determine whether ATF4 might be the mediator of the Nox4-dependent increase in CSE transcription, HUVECs were transduced with AdNox4 (or β-gal control virus) in the presence or absence of siRNA targeted to ATF4. Ectopic expression of Nox4 resulted in a dramatic increase in the level of ATF4 protein expression (Fig. 3*B*), concomitant with the increase in CSE mRNA expression observed. Crucially, silencing of ATF4 completely ablated the Nox4-dependent increase in CSE mRNA levels (Fig. 3*C*). Taken together these data suggest that ATF4 is both necessary and sufficient to mediate the Nox4-directed increase in CSE transcription in HUVECs.

We sought to determine whether the expression of ATF4 is similarly up-regulated in CMECs isolated from eNox4 Tg mice. However, ATF4 is a critical regulator of an integrated stress-induced response, and its sustained hyperactivation, due to unresolved stress, acts to up-regulate death effectors and directs the cell to an apoptotic fate (for review see Ref. 28). As a consequence the stability of ATF4 protein is subject to tight regulation by proteasomal degradation, mediated by at least two distinct mechanisms (29, 30). Perhaps accordingly, *in vivo* the “steady state” levels of ATF4 protein were not significantly higher in CMECs isolated from eNox4 Tg compared with WT mice, although a trend toward an increase was observed (Fig. 3*D*).

##### Nox4 Overexpression Acts to Phosphorylate eIF2α as a Cellular Stress Response

**FIGURE 4.**
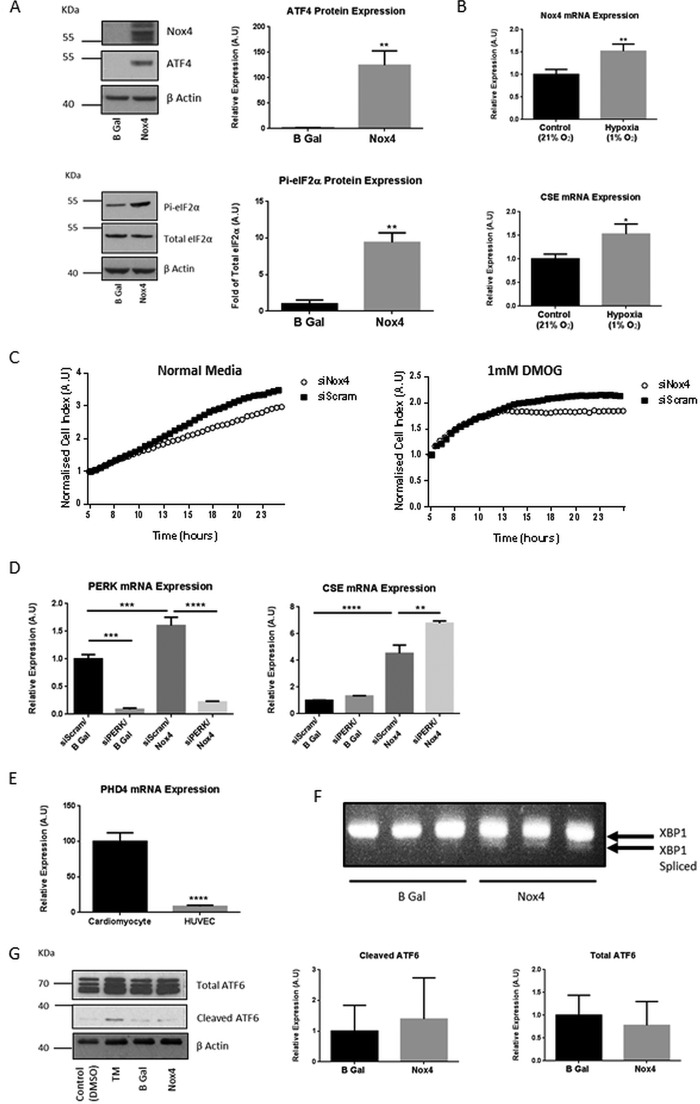
**Nox4 elicited a cellular stress response.** *A*, representative Western blots and quantitative densitometric analysis of ATF4, total eIF2α and phosphorylated eIF2α protein (*Pi-eIF2*α) in HUVECs after 24-h Nox4 or β-gal (*B Gal*) overexpression. *A.U.*, absorbance units. *B*, QPCR analyses of Nox4 and CSE mRNA expression in HUVEC after 1 h of incubation under atmospheric (*Control* (21%)) or hypoxic (*Hypoxia* (1% O_2_) conditions. *C*, HUVEC proliferation assessed after treatment with siRNA targeted to Nox4 (*siNox4*) or control siRNA (*siScram*) for 24 h. Cells were then seeded at equal densities onto E-plates (*ACEA*), and respective cell index was subsequently measured on an xCELLigence real time cell analyzer for 24 h. Cells were cultured in normal media or in media supplemented with 1 mm dimethyloxalylglycine (*DMOG*), administered 5 h after plating. The cell indices were normalized at the time point of compound administration in all cases (5 h). *D*, QPCR analyses of PERK and CSE mRNA expression in HUVECs after 48 h of treatment with siRNA targeted to PERK (*siPERK*) or control siRNA (*siScram*) together with 24 h Nox4 or β-gal overexpression as indicated. *E*, QPCR analyses demonstrating relative PHD4 mRNA expression in (rat) neonatal cardiomyocytes and HUVEC. *F*, PCR analysis of spliced XBP1 mRNA in HUVEC splicing after 24-h Nox4 or β-gal overexpression as indicated. *G*, representative Western blot and corresponding densitometric analysis for cleaved and total ATF6 protein expression in HUVECs after 24-h Nox4 or β-gal overexpression as indicated. HUVECs treated with tunicamycin (*TM*; 2 μg/ml) for 2 h compared with control (vehicle; DMSO) served as a positive control. All data were normalized to β-actin mRNA or protein expression apart from protein that was normalized to total eIF2α protein. *n* = 3 in all cases, *, *p* < 0.05; **, *p* < 0.01; ***; *p* < 0.001; ****, *p* < 0.0001.

The activation of ATF4, in response to multiple cellular stresses, is known to be regulated in part through the translational control of its mRNA. This mechanism has been extensively characterized and is reliant upon the phosphorylation status of the eIF2α GTP-binding protein (31). Phosphorylation of eIF2α at serine 51 attenuates global translation but permits ATF4 translation through a mechanism involving delayed ribosome reinitiation at an upstream inhibitory open reading frame that would in non-stressed cells block ATF4 expression (32). We assessed eIF2α phosphorylation at serine 51 after Nox4 overexpression in HUVEC lysates and found it significantly increased, concomitant with increased ATF4 protein expression (Fig. 4*A*).

The phosphorylation of eIF2α and the up-regulation of ATF4 expression are suggestive of a role for Nox4 as a mediator of a cellular stress response. Consistent with this, Nox4 expression has previously been shown to be increased in response to hypoxia in pulmonary arterial smooth muscle cells both *in vitro* and *in vivo* (33, 34). Accordingly, we found that Nox4 expression in HUVECs was similarly rapidly and robustly increased in response to hypoxia and that this increase in Nox4 expression correlated with a significant increase in CSE mRNA expression (Fig. 4*B*). Furthermore, siRNA-mediated ablation of Nox4 expression acted both to reduce the rate of proliferation of HUVECs at baseline, as demonstrated previously in pulmonary arterial smooth muscle cells (34), and significantly reduce the viability of HUVECs in response to administration of dimethyloxalylglycine, a chemical inducer of hypoxic signaling. This is evidenced by the dramatic decrease in growth rate seen in the Nox4 siRNA-treated HUVECs compared with control siRNA-treated cells (Fig. 4*C*). Taken together, these data suggest that increased Nox4 expression is a cellular response to hypoxia-mediated stress that is important to maintain cell viability.

The control of eIF2α phosphorylation is elicited by four eIF2α kinases that are activated in response to different stress stimuli (35). One of these kinases, PERK, is resident within the ER and is a mediator of one component of the unfolded protein response, which is activated in response to an accumulation of misfolded proteins (for review see Ref. 36). Nox4 is known to also be localized within the ER in HUVECs (37) (and other cells), whereas in cardiomyocytes, autophagy induced by energy stress has been shown to be mediated by Nox4-dependent activation of PERK-dependent signaling (27). Perhaps unexpectedly, silencing of PERK did not reduce the Nox4-induced increase in CSE expression and in fact resulted in increased expression (Fig. 4*D*). In cardiomyocytes, this activation of PERK by Nox4 was shown to be mediated via suppression of prolyl hydroxylase 4 (PHD4). We assessed the mRNA expression levels of PHD4 in HUVECs compared with rat cardiomyocytes and found PHD4 to be highly significantly less abundant in the endothelial cells (Fig. 4*E* and see “Discussion”). We additionally investigated whether Nox4 overexpression acted to promote other arms of the unfolded protein response in HUVEC. AdNox4 transduction of HUVEC resulted in no change in cleaved ATF6 levels and caused a minor increase in spliced XBP-1 (Fig. 4, *F* and *G*).

##### Nox4 Signals through the HRI/eIF2a/ATF4 Signaling Module to Increase CSE Transcription

**FIGURE 5.**
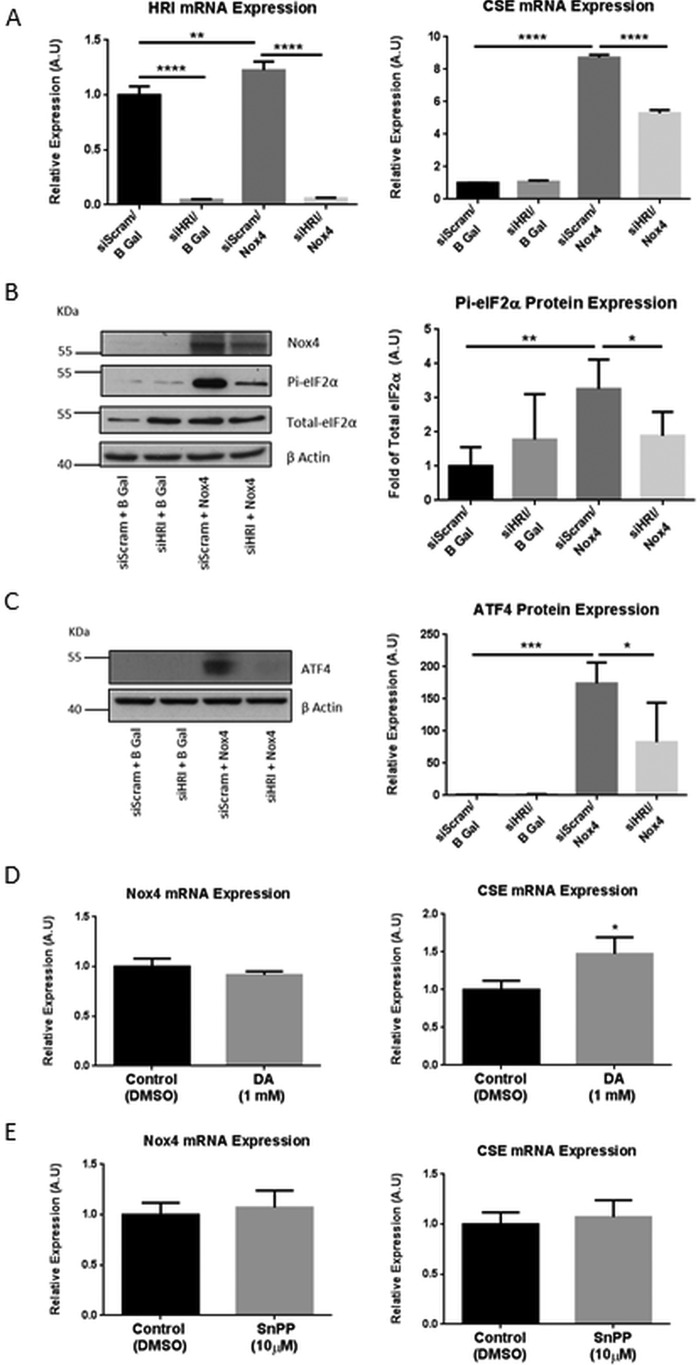
**Nox4 regulated CSE transcription through the HRI/eIF2α/ATF4 signaling module.** *A*, QPCR analyses of HRI and CSE mRNA expression in HUVEC after 48 h of treatment with siRNA targeted to HRI (*siHRI*) or control siRNA (*siScram*) together with 24-h Nox4 or β-gal overexpression as indicated. *A.U.*, absorbance units. *B*, representative Western blot analyses of Nox4, total eIF2α, and phosphorylated eIF2α protein (*Pi-eIF2*α) and quantitative densitometric analyses of Pi-eIF2α, normalized to total eIF2α phosphorylation, after treatments as in *A. C*, representative Western blot analyses and quantitative densitometric analyses of ATF4 protein expression after treatments as in *A. D* and *E*, QPCR analyses of Nox4 and CSE mRNA expression in HUVEC after inhibition of heme biosynthesis with 1 mm 4,6-dioxoheptanoic acid (*DA*) (*D*) or inhibition of heme oxygenase-1 with 10 μm tin protoporphyrin (*SnPP*) (*E*) for 24 h. All data were normalized to β-actin mRNA and protein expression apart from *B. n* = 3 for mRNA analyses, *n* = 5 for protein analyses. *, *p* < 0.05; **, *p* < 0.01; ***; *p* < 0.001; ****, *p* < 0.0001.

Another stress-responsive kinase that acts to phosphorylate eIF2α and that has also been shown to be up-regulated in response to ROS is the HRI (38). By contrast to the silencing of PERK, siRNA-mediated depletion of HRI resulted in a highly significant reduction in CSE mRNA levels after Nox4 overexpression (Fig. 5*A*). In the same experiment we further observed a significant decrease in both eIF2α phosphorylation and ATF4 protein expression upon silencing of HRI in the presence of Nox4 overexpression (Fig. 5, *B* and *C*). Taken together these data demonstrate a role for the HRI/eIF2α/ATF4 signaling module in the Nox4-induced regulation of CSE expression in endothelial cells and suggest the potential involvement of heme bioavailability in the regulation of CSE. To deplete or enhance intracellular heme levels, HUVECs were treated with an inhibitor of heme biosynthesis (4,6-dioxoheptanoic acid) or an inhibitor of heme oxygenase (tin protoporphyrin IX dichloride), respectively. Administration of 4,6-dioxoheptanoic acid acted to increase CSE mRNA levels significantly, whereas administration of tin protoporphyrin did not affect CSE expression. Nox4 mRNA levels were not altered in either case (Fig. 5, *D* and *E*). These data are consistent with heme insufficiency being an activator of CSE transcription in HUVECs and suggest that Nox4 may act upstream of heme depletion in this scheme.

##### ATF4 Activates CSE Transcription via the Cis-regulatory Sequence(s) within the First Intron

**FIGURE 6.**
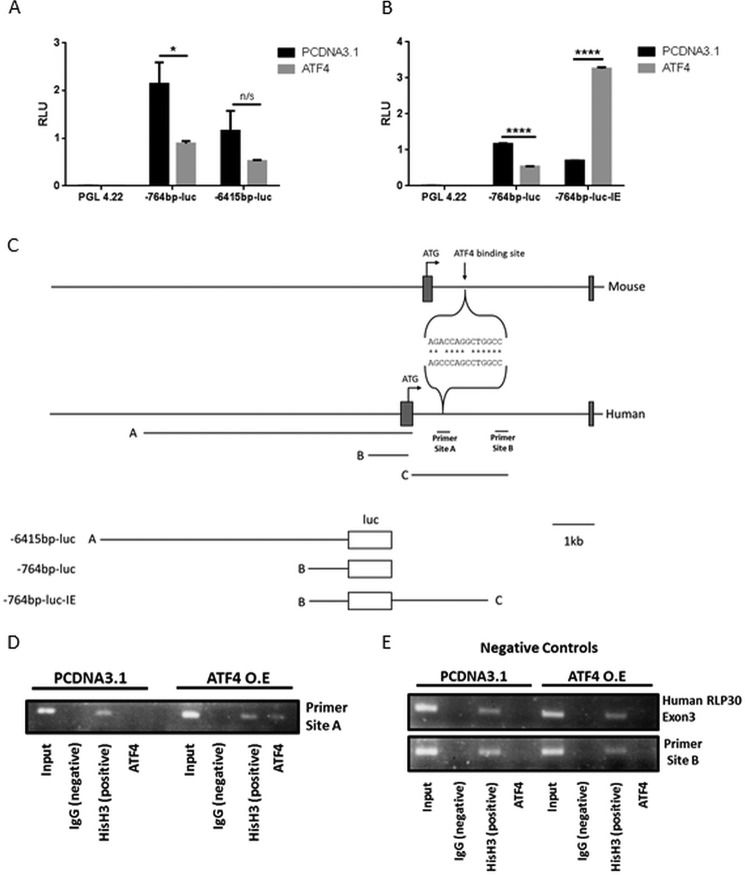
**ATF4 induced CSE transcription via direct binding to a cis regulatory intronic site.** *A* and *B*, luciferase activity resulting from HEK cells transfected with constructs as indicated together with (empty vector control plasmid) pCDNA3.1 or overexpressed ATF4. *RLU*, relative light units. *n* = 4. *, *p* < 0.05; ****, *p* < 0.0001. *n/s*, not significant. *C*, schematic representation of alignment of putative ATF4-binding sites within intron 1 of mouse and human CSE gene loci. The genomic fragments contained in each luciferase construct are indicated. *D* and *E*, formaldehyde cross-linked chromatin prepared from HEK cells transfected with pCDNA3.1 or ATF4 incubated with normal rabbit IgG (negative control), anti-acetyl-histone H3 (positive control), or anti-ATF4 as indicated. Aliquots of chromatin before immunoprecipitation served as a positive control (*input*). Purified DNA was analyzed using primers specific for site A or site B as indicated in *panel C* or exon 3 of Human RLP30. The results presented are representative of three separate experiments.

To determine how ATF4 regulates CSE expression, we first cloned an ∼6.5-kb human genomic fragment comprising the 6415-bp proximal promoter sequence upstream of the CSE transcriptional start site in addition to the CAP site and 191-bp 5′ untranslated region (UTR) into a promoterless luciferase reporter gene vector (pGL4.22; Fig. 5*C*). We assessed the ability of this fragment to mediate transcriptional transactivation by ATF4 by transient transfections into HEK cells, which (by contrast to HUVEC cells) are readily and efficiently transfected. The 6.5-kb CSE promoter fragment-containing construct (−6415bp-luc) directed high levels of luciferase expression compared with the promoterless pGL4.22 control vector, demonstrating promoter activity (Fig. 6*A*). However, this construct displayed no increase in luciferase activity upon co-transfection with an ATF4-overexpressing vector (rather expression was decreased), suggesting that the *cis*-regulatory sequences that mediate ATF4 transactivation are not located within this proximal promoter region (Fig. 6*A*). A genome-wide chromatin immunoprecipitation-sequencing (ChIP-seq) analysis of ATF4-binding sites within mouse embryonic fibroblasts, upon induction of ER stress by tunicamycin, has been reported (39). One of the sites identified in this study maps to the mouse CSE gene locus, at position +938 (relative to the transcriptional start site) within the first intron (Fig. 6*C*). Intriguingly, although ATF4 ChIP-seq data does not appear to be available for the human genome, sequences within the first intron are indicated as being potentially regulatory due to sequence and/or altered chromatin structure on the ensemble genome browser. We, therefore, sought to test whether the sequence within intron 1 of the human CSE gene might mediate the ATF4 transcriptional transactivation. Thus we generated a “basal” CSE promoter-reporter gene comprising a 5′ deletion of −6415-luc, which contained only the 764-bp sequence upstream of the CAP site in addition to the UTR (−764bp-luc; Fig. 5*C*). Transfection of this construct into HEK cells again resulted in high levels of luciferase activity compared with those directed by pGL4.22, but once more activity was decreased upon ATF4 co-expression (Fig. 5*A*). A 2.4-kb genomic fragment, immediately downstream of exon 1, that comprises potentially regulatory intronic sequence (as indicated in Ensembl) was subsequently tested for “enhancer” function. It was cloned downstream of the polyadenylation site of the luciferase gene, within the basal promoter construct, −764bp-luc, to generate −764bp-luc-IE (Fig. 6*C*). By contrast to −764bp-luc, upon co-transfection with ATF4 the activity of −764bp-luc-IE was dramatically increased, indicating that sequences within this intronic fragment mediate the ATF4-dependent regulation of CSE transcription (Fig. 6*B*).

##### ATF4 Binds Directly to an Intronic Sequence within the Human CSE Gene

To determine whether the transcriptional up-regulation mediated by ATF4 results from direct binding of ATF4 to sequences within the first intron of the CSE gene, we performed ChIP analyses. ATF4 is a member of the ATF/CREB (cAMP-response element-binding protein) family of basic region-leucine zipper (bZip) transcription factors that bind the CRE (cyclic AMP response element) DNA consensus sequence, TGACGTCA. bZip transcription factors bind DNA as dimers via their leucine zipper domains, and ATF4 is known to have numerous potential dimerization partners that can significantly change the DNA binding site compared with that of the parental homodimer (40). The ATF4 binding region, identified by ChIP-seq, within the first intron of the mouse CSE gene does not comprise a canonical ATF4 binding motif. To determine the likely ATF4-binding site within the human intronic sequence, we compared a short sequence (60 bp) comprising the known mouse ATF4 binding sequence to the 2.4-kb human intronic *cis*-regulatory sequence using an online alignment tool, Clustal Omega. This identified a short region of significant homology (12/14-bp identity) that mapped from 822 to 835 bp of the human CSE intron 1 (Fig. 6*C*). We designed primers spanning this region to test for ATF4 binding in the ChIP analyses (*site A*, Fig. 5*C*). HEK cells were transfected with −764-luc-IE with or without co-transfected ATF4, and chromatin was prepared for analysis. We demonstrated previously that transfected plasmid is assembled into chromatin and is, therefore, suitable for these analyses (41). We demonstrated specific binding of ATF4 to this region of the human CSE intron upon ATF4 overexpression (Fig. 6*D*). By contrast, a region at the 3′ end of the 2.4-kb *cis*-regulatory intronic fragment (site B, Fig. 6*C*) did not bind ATF4 either in control or ATF4-overexpressing HEK cells (Fig. 6*E*). In addition, negative control primers (provided in the ChIP kit) did not demonstrate ATF4 binding to endogenous HEK chromatin (Fig. 6*E*). As expected, all genomic regions were shown to bind to histone H3, and specificity of binding was further demonstrated as no region bound to normal rabbit IgG (Fig. 6, *D* and *E*).

## Discussion

The data presented here shed light upon the molecular mechanisms within the endothelium which underlie the regulation of expression of the enzyme CSE and thus potentially modulate the generation of the gasotransmitter, H_2_S. Because of its gaseous and highly reactive nature, the production of H_2_S needs to be tightly controlled. It is known that the activity of CSE is regulated by calmodulin and intracellular calcium levels (7). The data presented here delineate a molecular pathway that regulates CSE expression through a tightly regulated response to Nox4-dependent redox signaling. We demonstrate that CSE expression is strongly inducible within endothelial cells, in response to increased expression of Nox4. Nox4 requires only p22^phox^ as an obligate partner for its activity (20), and the protein expression levels of these subunits in HUVECs suggest that Nox4 is the limiting factor for this activity. In addition, the increased CSE transcription upon Nox4 overexpression in HUVECs was shown to be both time- and dose-dependent, supporting a role for Nox4 levels, and hence activity, in the regulation of H_2_S generation. We also demonstrate that Nox4 is indeed a physiological regulator of CSE expression in HUVECs, as CSE expression decreased upon siRNA-mediated silencing of either Nox4 or p22^phox^. The effect of p22^phox^ silencing upon CSE expression was, in fact, more pronounced than that of silencing Nox4 in these experiments. Therefore, although our data support the role of Nox4 as a cellular source of ROS that mediates the redox-dependent regulation of CSE, they do not exclude the involvement of other cellular sources of ROS, including other NADPH oxidase isoforms (the lower expression of other isoforms, Nox1 and Nox2, within HUVEC notwithstanding).

### 

#### 

##### Regulation of Vascular Tone by Nox4 Is Inhibited by PPG

The effect of endothelial-specific ectopic expression of Nox4 was investigated *in vivo* in our eNox4 Tg mice. CMECs isolated from these mice displayed higher levels of CSE mRNA and protein than their WT littermates. CSE is believed to play a critical role in the maintenance of vascular tone via the generation of H_2_S (5). We studied the vascular function of the eNox4 Tg mice by wire myography. We observed that aortic vessels isolated from these mice displayed a hypo-contractile phenotype compared with WT controls, in response to PE, in accordance with a previous report (16). In the presence of PPG, an inhibitor of CSE, this difference in contraction to PE was ablated, consistent with a functional role of the increased CSE expression (and hence potentially H_2_S production) in this phenotype. However, at the concentration used in these experiments (20 mm), PPG acts as an inhibitor of other pyridoxal-5′-phosphate-dependent enzymes (42). To demonstrate definitively the involvement of CSE in the Nox4-dependent regulation of vascular tone, it will be necessary in the future to cross our eNox4 Tg mice to a line in which CSE has been genetically ablated. In previous studies, however, we demonstrated increased relaxation to acetylcholine in aortic rings isolated from these mice that was ablated by the addition of high extracellular potassium or by inhibitors of K_Ca_ channels (15), suggesting that an endothelium-derived hyperpolarizing factor-like activity contributes to Nox4-dependent increased vasodilatation in this setting. Although H_2_O_2_ itself can act as an endothelium-derived hyperpolarizing factor (43), H_2_S is also believed to cause vasodilatation through hyperpolarization (4). Our current results might, therefore, suggest that Nox4-dependent H_2_S production may be an important contributor to this increased dilatation. Further experiments, however, will need to be performed using resistance arteries, in which the hyperpolarization effects of H_2_S are mainly associated to determine whether Nox4 is exerting its vasodilationary effects via increased H_2_S production.

##### Nox4 Promotes CSE Transcription via the HRI-eIF2α-ATF4 Pathway

**FIGURE 7.**
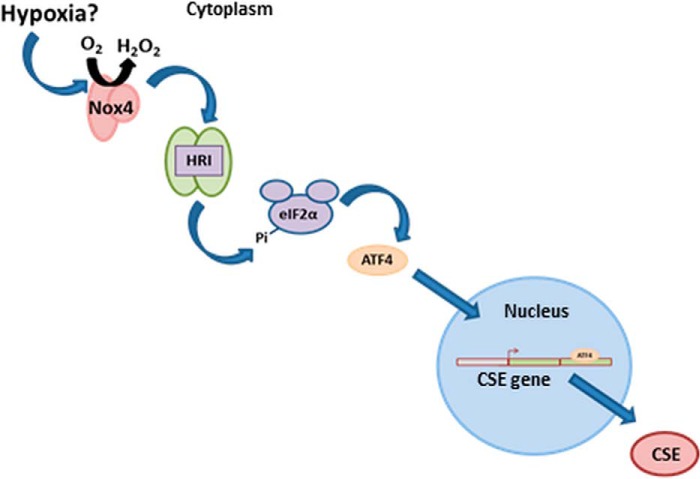
**A schematic illustration of the mechanism underscoring the Nox4-induced increase in CSE expression in endothelial cells.** Hypoxia potentially acts as an upstream signal to induce Nox4 expression. Enhanced Nox4 activity leads to HRI activation and subsequent phosphorylation of eIF2α on serine 51. eIF2α phosphorylation attenuates global translation but permits ATF4 protein expression. ATF4 then binds to an intronic enhancer element in intron 1 of the CSE gene and subsequently promotes CSE expression.

We elucidate here a molecular pathway that links Nox4-dependent redox signaling to activation of HRI kinase and subsequent accumulation of the transcription factor ATF4 via phosphorylation of eIF2α. We also show that CSE is a direct transcriptional target of ATF4 and that the rate of CSE transcription is increased by binding of ATF4 to *cis*-regulatory sequence(s) within its first intron (Fig. 7). The finding that Nox4 promotes the activation of the integrated stress response in endothelial cells via the HRI kinase was unexpected. Previous studies in cardiomyocytes have shown that Nox4 is induced in response to glucose deprivation and acts to promote autophagy via activation of the eIF2α-ATF4 pathway via promotion of PERK activity (27). The molecular target of ROS-dependent oxidation in this case was suggested to be the PERK inhibitor, PHD4, within the ER. The activation of the eIF2α-ATF4 pathway by Nox4 described here in endothelial cells, however, was found not to be dependent upon PERK. In fact, silencing of PERK acted to increase CSE mRNA levels upon overexpression of Nox4. A possible explanation for the lack of activation of PERK by Nox4 in these experiments might be the significantly lower levels of PHD4 expression that are observed in HUVECs compared with cardiomyocytes (Fig. 4*E*). However, given the known localization of Nox4 within the ER in endothelial cells (37) we assessed whether there was a general effect of Nox4 on the unfolded protein response, which involves the ATF6 and XBP-1 limbs, in addition to the ATF4 arm (36). Although there was a minor increase in spliced XBP-1 upon Nox4 overexpression, we observed no change in cleaved ATF6 levels, suggesting that the effect of Nox4 to increase CSE expression is specific to the ATF4 arm.

By contrast to the genetic silencing of PERK, siRNA-mediated ablation of HRI resulted in a significant decrease in CSE transcription upon Nox4 overexpression. The function of HRI has predominantly been studied in erythroid cells, where it plays a crucial role in cell survival in situations of iron/heme deficiency (44). The redox-dependent mechanism that underlies the activation of this kinase is not currently understood, although previous studies in both erythroid cells and mouse embryonic fibroblasts have reported its activation independent of heme via “oxidative stress” induced by exposure to heavy metals such as arsenite (38, 45) or lead (46). It is, however, also possible that the ROS generated by Nox4 acts to deplete heme levels (and so activate the kinase) either directly or indirectly. Thus heme is known to be subject to oxidative degradation by H_2_O_2_ (47), whereas the enzymatic degradation of heme by heme oxygenase-1 (HO-1) may be promoted by Nox4 in endothelial cells. HO-1 is a well known transcriptional target of the transcription factor, Nrf2, which we have demonstrated previously to be activated in response to increased Nox4-generated ROS in cardiomyocytes (23).

In support of a role for heme in the regulation of CSE expression, we demonstrate here that heme depletion via inhibition of heme synthesis also resulted in a significant increase in CSE expression (Fig. 5*D*). It is perhaps intriguing that heme levels might be a regulator of CSE expression and hence H_2_S production in endothelial cells. Thus the other gasotransmitters, which are generated within the endothelium to regulate vascular tone, NO and CO, both require heme for their generation; endothelial nitric-oxide synthase binds heme within its oxygenase domain (48) and HO requires heme as its substrate. There are considerable functional and regulatory relationships between the three gasotransmitters (49, 50), and therefore, it is plausible that H_2_S production might need to be increased under situations where the bioavailability of NO and CO become limited due to low heme levels.

##### Nox4 as a Mediator of Cellular Homeostasis

Unlike other NADPH oxidases, the activity of Nox4 is predominantly regulated at the gene expression level rather than by post-translational mechanisms (51). Consistent with its role in cellular homeostasis, many studies have demonstrated this expression to be induced by diverse cellular stresses including nutritional stress, pressure-overload induced cardiac hypertrophy, and, perhaps most relevant to vascular endothelial cells, hypoxia (33, 52, 53). We confirmed the increase in Nox4 expression in HUVECs upon cellular exposure to hypoxia and further demonstrated that this increase in Nox4 expression is concomitant with an increase in CSE transcription and acts to maintain cell viability (Fig. 4, *B* and *C*). In addition to vascular tone, endothelial-derived H_2_S has increasingly been implicated in angiogenesis (54) and vascular remodeling after ischemic injury (55), whereas several studies have shown Nox4 to promote angiogenesis in response to hypoxic/ischemic insult (16, 17, 56). Thus components of the molecular pathway described here may prove useful therapeutic targets in the future treatment of both hypertension and vascular disease states such as critical limb ischemia.

In addition to its function as a generator of H_2_S within the vasculature, CSE is also a critical enzyme in the trans-sulfuration pathway, which converts homocysteine to cysteine. This pathway is particularly important in the liver and kidney where its regulation is critical in the maintenance of appropriate levels of the critical redox buffer, glutathione, and hence cellular redox homeostasis (57). Moreover, mis-regulation of the trans-sulfuration pathway impacts upon plasma homocysteine levels and multiple associated pathologies (57). Nox4 is highly expressed in both liver and kidney, and a recent genome wide association study identified a significant association between Nox4 and aberrant homocysteine levels (58). We, therefore, suggest that the potential role of Nox4 in the regulation of the trans-sulfuration pathway in relevant biological systems may be an important field of future investigation.

To conclude, we have identified a molecular pathway that links an inducible mediator of redox signaling pathways (Nox4) to an important enzymatic activity that plays critical roles in vascular homeostasis (CSE). A better understanding of the molecular mechanisms that regulate the transcriptional induction of Nox4 in vascular cells is now needed.

## Author Contributions

R. K. M. designed and performed all experiments and co-wrote the paper. A. C. B. conceived and coordinated the study and co-wrote the paper. A. M. S., C. S., and P. E. assisted with interpretation of data. T. V. A. M. gave experimental guidance and assisted in interpretation of results. O. P. gave guidance and assisted in experiments described in Fig. 2. D. M. gave technical assistance. J. R. B. gave experimental guidance for Fig. 4. All authors reviewed the results and approved the final version of the manuscript.

## Footnotes

2 The abbreviations used are:

CSE: cystathionine γ-lyase
HUVEC: human umbilical vein endothelial cell
ROS: reactive oxygen species
Nox4: NADPH oxidase 4
Tg: transgenic
eNox4 Tg: Nox4-overexpressing transgenic
ATF4: activating transcription factor 4
HRI: heme-regulated inhibitor kinase
PERK: protein kinase R-like ER kinase
HBSS: Hanks' balanced salt solution
QPCR: quantitative PCR
PPG: propargylglycine
CMEC: cardiac microvascular endothelial cell
ER: endoplasmic reticulum
PHD4: prolyl hydroxylase 4
ChIP-seq: immunoprecipitation-sequencing
PE: phenylephrine.
